# TLR Accessory Molecule RP105 (CD180) Is Involved in Post-Interventional Vascular Remodeling and Soluble RP105 Modulates Neointima Formation

**DOI:** 10.1371/journal.pone.0067923

**Published:** 2013-07-02

**Authors:** Jacco C. Karper, Mark M. Ewing, Margreet R. de Vries, Saskia C. A. de Jager, Erna A. B. Peters, Hetty C. de Boer, Anton-Jan van Zonneveld, Johan Kuiper, Eric G. Huizinga, T. Harma C. Brondijk, J. Wouter Jukema, Paul H. A. Quax

**Affiliations:** 1 Department of Surgery, LUMC, Leiden, The Netherlands; 2 Einthoven Laboratory for Experimental Vascular Medicine, LUMC, Leiden, The Netherlands; 3 Department of Cardiology, Leiden University Medical Center, LUMC, Leiden, The Netherlands; 4 Department of Biopharmaceutics, Leiden University, The Netherlands; 5 Department of Nephrology, Leiden University Medical Center, LUMC, Leiden, The Netherlands; 6 Crystal and Structural Chemistry, Bijvoet Center for Biomolecular Research, Department of Chemistry, Utrecht University, Utrecht, The Netherlands; University of Illinois College of Medicine, United States of America

## Abstract

**Background:**

RP105 (CD180) is TLR4 homologue lacking the intracellular TLR4 signaling domain and acts a TLR accessory molecule and physiological inhibitor of TLR4-signaling. The role of RP105 in vascular remodeling, in particular post-interventional remodeling is unknown.

**Methods and Results:**

TLR4 and RP105 are expressed on vascular smooth muscle cells (VSMC) as well as in the media of murine femoral artery segments as detected by qPCR and immunohistochemistry. Furthermore, the response to the TLR4 ligand LPS was stronger in VSMC from RP105^−/−^ mice resulting in a higher proliferation rate. In RP105^−/−^ mice femoral artery cuff placement resulted in an increase in neointima formation as compared to WT mice (4982±974 µm^2^ vs.1947±278 µm^2^,p = 0.0014). Local LPS application augmented neointima formation in both groups, but in RP105^−/−^ mice this effect was more pronounced (10316±1243 µm^2^ vs.4208±555 µm^2^,p = 0.0002), suggesting a functional role for RP105. For additional functional studies, the extracellular domain of murine RP105 was expressed with or without its adaptor protein MD1 and purified. SEC-MALSanalysis showed a functional 2∶2 homodimer formation of the RP105-MD1 complex. This protein complex was able to block the TLR4 response in whole blood ex-vivo. In vivo gene transfer of plasmid vectors encoding the extracellular part of RP105 and its adaptor protein MD1 were performed to initiate a stable endogenous soluble protein production. Expression of soluble RP105-MD1 resulted in a significant reduction in neointima formation in hypercholesterolemic mice (2500±573 vs.6581±1894 µm^2^,p<0.05), whereas expression of the single factors RP105 or MD1 had no effect.

**Conclusion:**

RP105 is a potent inhibitor of post-interventional neointima formation.

## Introduction

In interventional cardiology restenosis remains a critical determinant of long-term efficacy of Percutaneous Coronary Interventions (PCI). Neointima formation is a common feature of restenosis and atherosclerosis and is characterized by proliferation and migration of vascular smooth muscle cells (VSMC) and extracellular matrix formation [1], [2]. These processes are strongly mediated by inflammation and influx of inflammatory cells in the affected vessel wall [3]. Under hypercholesterolemic conditions this is accompanied by lipid accumulation in the vessel wall, thereby initiating a process of accelerated atherosclerosis. Previously, we and others described an important causal role for Toll Like Receptor 4 (TLR4) in neointima formation. Furthermore, TLR4 activation by local application of LPS had a stimulatory effect on neointima formation and accelerated atherosclerosis development[4]–[6]. LPS initiates TLR4 activation and the resulting inflammatory reaction causes a release of many pro-inflammatory cytokines that will affect the pathophysiological process of neointima formation strongly [7], [8]. TLR4 is expressed on VSMC and is involved in their proliferation [9]. Previously, we were able to detect co-localization between endogenous TLR4 ligands (HSP60 and fibronectin-EDA) and TLR4 during vascular remodeling [4], [6].

TLR4 is a pattern recognition receptor (PRR) of the innate immune system and is expressed by both immune and non-immune cells. It is the most robust and complex signaling TLR and can be activated by an array of ligands, including damage associated molecular patterns (DAMPs), which can for instance be degradation products of endogenous matrix molecules, necrotic cells, oxidized molecules and inflammation-specific molecules [10]. Activation of TLR4 by binding of LPS and or other ligands that become available after cell stress or tissue damage [11], [12] is dependent on the presence of MD2, a TLR accessory molecule.

It is becoming more and more clear that accessory molecules play a key role in the complex TLR-signaling. Accessory molecules can be divided into molecules that reside in the endoplasmic reticulum, molecules that directly interact with TLR ligands or regulatory molecules present on the cell surface such as RP105 (CD180) [13]. RP105 is reported to be a physiological TLR4 inhibitor on myeloid cells.

In the presence of RP105 on myeloid cells activation of the TLR4 signaling pathway is directly reduced making RP105 one of the most important accessory molecules acting as a regulator of TLR4 signaling. Structurally, RP105 is highly homologous to TLR4, but lacks the intracellular Toll Interleukin Receptor (TIR) domain. TLR4 activation in cellular immunity, induced by e.g. its ligand LPS, is negatively regulated by RP105[14]–[16]. RP105 forms a complex with MD1, a MD2 homolog, in an unusual 2∶2 homodimer [17], [18]. This orientation is opposite to that of known ligand-induced TLR-homodimers. The functional consequence of this unusual dimer configuration is still under debate [17], [18]. RP105/MD1 is thought to bind LPS and other TLR4 ligands, therefore RP105 is linked to several inflammatory processes including autoimmune diseases [16], [19], [20]. So far, the involvement and possible causal role of RP105 in vascular remodeling and other cardiovascular related events is unknown.

In the current study we demonstrate a functional role for RP105 in vascular remodeling during neointimal formation by the use knockout mice, cultured VSMC, purified proteins and by in vivo gene transfer mediated overexpression of soluble RP105 (solRP105) protein combined with the MD1 accessory protein.

## Materials and Methods

### Cell Culture and Cell Culture Immunostaining

Murine VSMCs explanted from aortas were cultured and incubated overnight with either PBS or LPS (1 ng/ml or 10 ng/ml). Cells were either cultured for RNA isolation or were cultured on glass coverslips and fixed. The latter was followed by blocking with PBS, 3% BSA, 2% FCS for one hour at room temperature. After blocking, primary antibodies and isotype controls were incubated for one hour followed by washing and incubation with labeled secondary antibodies for one hour in the dark. TLR4 presence was demonstrated using TLR4 antibody (rabbit anti-human, Santa Cruz). RP105 was stained with RP105 antibody (rat anti-mouse, Abcam, United Kindom). A ZEISS 510 microscope was used for analysis and confocal microscopy.

#### Macrophage

Macrophages were derived from bone marrow from tibia and femur and seeded at a density of 500.000 cells/well in 6-wells plates and cultured for 7 days in RPMI GlutaMax (Gibco) supplemented with 100 U/ml penicillin/streptavidin, 25% Fetal Calf Serum (FCS) and 20 µg/ml M-CSF (Myltec Biotechnologies) as described previously [21].

#### Dendritic cells

Dendritic cells were derived from bone marrow from tibia and femur and seeded at a density of 1.500.000 cells/well in 6-wells plates and cultured for 10 days in RPMI Glutamin (Gibco) supplemented with 100 U/ml penicillin/streptavidin, 10% Fetal Calf Serum (FCS) and 20 µg/ml GM-CSF (Myltec Biotechnologies). as described previously [22]. Cells were cultured in the presence of 1 ng/ml LPS.

### RT-PCR

From VSMCs, total RNA was isolated using Tri-Reagent (Sigma-Aldrich) according to the manufacturer's protocol. The expression levels of TLR4 and RP105 were analyzed by real time polymerase chain reaction (RT-PCR) (Taqman, Applied Bioscience) on non-stimulated VSMCs and VSMCs stimulated with LPS overnight. The relative mRNA expression levels were determined using GAPDH as housekeeping gene and the 2[−ΔΔC(T)] method. Values were expressed as fold of unstimulated controls.

### SMC Proliferation Assay

Murine SMCs, explanted from aortas, were subsequently cultured, characterized, and proliferation was measured using the 3H-Thymidine incorporation method as described previously [23]. Briefly, cells were seeded in a 24 well disk at a density of at least 100.000 cells per cm^2^. Next, cells were made quiescent for 48 hours in serum free medium followed by stimulation with LPS in full medium containing 10% FCS. Methyl-^3^H-thymidine incorporation (Amersham, 0.25 µCu per well) over a 16 hours period was measured by liquid-scintillation counting and compared to non stimulated control cells of WT and RP105^−/−^ VSMC in serum free medium. All experiments were done in triplicates.

### Animals

Animal experiments were approved by the Institutional Committee for Animal Welfare of the Leiden University Medical Center (LUMC) and were performed according the regulatory guidelines. 10 week old male wild type (WT, C57/B6) and 10 week old male RP105^−/−^ animals (C57/B6 background, backcrossed for more than 10 generations) bred in our laboratory, were used. RP105^−/−^ mice were provided by K.Miyake, Tokyo University, Japan and described previously [24]. 10 week old male hypercholesteremic ApoE3Leiden mice were used to study the effect of soluble RP105 overexpression. ApoE3Leiden mice received a western-type diet that started 3 weeks before surgery and continued during the experiment. One week before surgery cholesterol levels in serum were determined (Roche Diagnostics, The Netherlands). All mice received water and food ad libitum.

### Murine Model for Neointima Formation

Non-constricted polyethylene cuffs were placed around the femoral arteries as described previously [25] Mice were killed 3 weeks after cuff placement. The hypercholesterolemic mice were sacrificed after two weeks, since at this time point first signs of accelerated atherosclerosis and foam cells formation becomes detectable. For local TLR4 stimulation, TLR4 ligand LPS (1 µg/µL) (from Escherichia coli 055:B5, Sigma Aldrich, The Netherlands) was dissolved in pluronic gel (F-127, Sigma Aldrich, The Netherlands) and administered inside the cuff [4].

### Purification of solRP105 and solRP105-MD1

#### Plasmid constructs

The extracellular domain of mouse RP105 comprising residues Thr21-Ala630 was inserted into a pUPE expression vector (U-Protein Express BV, Utrecht) that includes an N-terminal (His)6-(StrepII)3-TEV-tag. Mouse MD1 without its native signal sequence; residues Asp20-Ser162, was cloned into a pUPE expression vector that includes an N-terminal (His)6-tag. Both vectors include the human cystatin-S signal peptide for efficient secretion and expression is controlled by the CMV-promoter. The cloning procedure introduces a triple-alanine sequence at the C-terminus of both proteins.

#### Protein expression and purification

Expression vectors encoding solRP105 and MD1 were transfected transiently, either individually or in combination, in HEK293-EBNA1-S cells. Culture supernatant was harvested 6 days post-transfection. Supernatant was concentrated 10-fold using a Quixstand hollow fiber system (GE Healthcare) prior to diafiltration into 50 mM Tris-Cl, pH 8.0, 300 mM NaCl. RP105 and RP105-MD1 complex were purified using Ni-Sepharose/Streptactin-Sepharose tandem-affinity-purification and eluted in 50 mM Tris-Cl, pH 8.0, 300 mM NaCl supplemented with 5 mM desthiobiotin. MD1 was purified by Ni-Sepharose affinity purification in 50 mM Tris-Cl pH 8.0, 300 mM NaCl supplemented with 250 mM imidazole followed by dialysis to buffer without imidazole. N-glycosylation of SDS-denatured MD1 was analysed by cleavage with PNGase (Roche) according to the manufacturer’s protocol. Protein quality was further assessed by size-exclusion chromatography on a Superdex 200 PC3.2/30 column (GE Healthcare) using 50 mM Tris-Cl, pH 8.0, 300 mM NaCl as the running buffer at 0.05 ml/min. The molecular weight of the solRP105-MD1 complex was determined by size exclusion chromatography with multi-angle light scattering (SEC-MALS) using a WTC-030S5 column (Wyatt technologies) and 50 mM Tris-Cl, pH 8.0, 300 mM NaCl as the running buffer at 0.5 ml/min. Molecular weight was calculated by ASTRA software version 5.3.4.18 Wyatt technologies).

### In vivo Overexpression of Soluble RP105

In vivo overexpression of soluble RP105 (solRP105) was achieved by gene-transfer of the expression plasmids encoding the extracellular domain of RP105, MD1 or both plasmids together. A Luciferase encoding plasmid was used as control. Electroporation mediated gene transfer was performed 3 days before cuff placement by injecting 50 µg of each plasmid per leg, either Luciferase, solRP105, MD1 or MD1 plus solRP105, into the calf muscles of both legs, followed by electroporation (8 pulses of 10 ms, field strength of 200 V/cm [Sq Wave Electroporator ECM 830, BTX] using Caliper Electrodes). Calf muscles were primed with an intramuscular injection containing 30 µL of hyaluronidase (0.45 U/µL, Sigma) one hour before electroporation as described previously [26].

### Whole Blood Assay

Blood from three C57/B6 mice was collected (180 µl blood with 20 µl heparin) and incubated with either solRP105 purified protein (7.5 µg/ml, MW = 74706), solRP105-MD1 purified protein complex (9 µg/ml, MW = 90975) or PBS. Blood was stimulated overnight with 100 ng/ml LPS and subsequently the cytokine TNFα was measured by ELISA (BD biosciences).

### Morphological Quantification

Mice were sacrificed 21d (or as stated otherwise) after surgery for histological analysis. Arterial segments were harvested after perfusion fixation (100 mm Hg) with 4% formaldehyde, fixed overnight and paraffin-embedded using an automated tissue processor (Leica, Germany). Cross-sections were made throughout the embedded cuffed arteries. Six representative sections per vessel segment were stained with Elastin von Giesson and Haematoxylin-Phloxine- Saffron (HPS) for histological and morphometric analysis (QWin, Leica, Germany). Neointima formation was defined as the area between lumen and the internal elastic lamina.

### Immunohistochemisty on Vessels

Paraffin embedded sections (5 µm) were de-paraffinized in xylene. Peroxidase activity was blocked by incubation in 0.3% (v/v) H_2_O_2_ in methanol for 20 min. Antigen retrieval was performed and tissue sections were pre-incubated with 5% bovine serum albumin (BSA), followed by overnight incubation with detecting antibody for either RP105 antibody (rat anti mouse, Abcam, United Kingdom) or CD45 (rat-anti mouse). After washing in PBS, sections were incubated for 1 h with a secondary antibody, washed in PBS, incubated for 1 h with AB complex (Vector laboratories, The Netherlands) and visualized with Novared (Vector laboratories) or DAB (Dako, Denmark). Slides were counterstained with haematoxylin. Immunopositive areas of leukocytes were calculated as percentage of total either media or neointima area in cross-sections by morphometry (Qwin, Leica, Germany). To confirm the specificity of the IHC staining, parallel sections were incubated with 1% PBS/BSA alone without adding the primary antibody or with IgG isotype control or without first antibody. Sections were incubated with the secondary antibody, AB complex and were visualized with Novared. Controls were all negative.

### Statistics

For the animal experiments, values are presented as mean ± standard error of the mean (SEM). Statistical significance was calculated in SPSS for Windows 17.0. Differences between groups were determined using a non-parametric Mann-Whitney test or One-Way ANOVA. In vitro data was statistically analyzed with a Student’s-T-test.

## Results

### RP105 and TLR4 are both Expressed by VSMC

**Figure 1:**
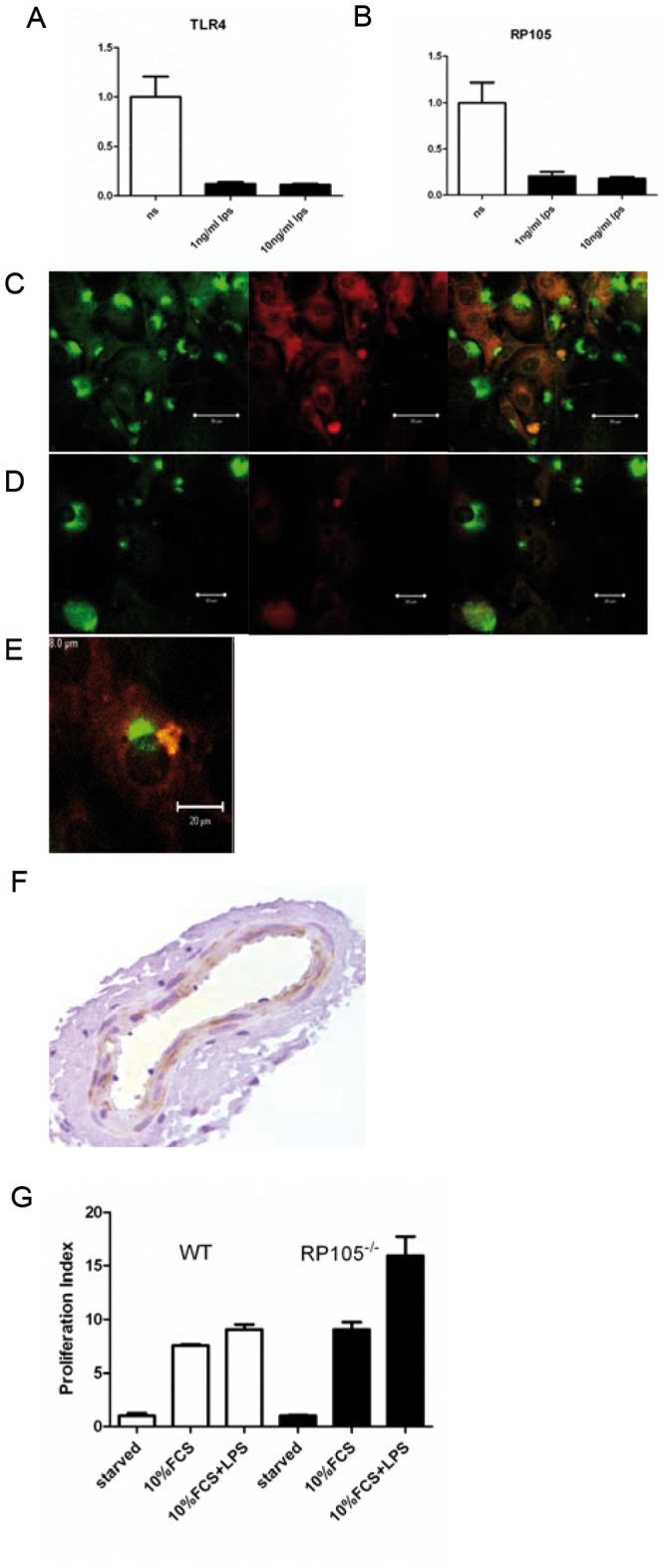
RT-PCR of TLR4 (A) or RP105 (B) on wild type VSMC that were incubated with PBS, 1 ng/ml LPS or 10 ng/ml LPS. Immunostaining of TLR4 (green) or RP105 (red) or both (overlay) on wild type VSMC that were incubated with PBS (C) or 1 ng/ml LPS. (D) Colocalized TLR4 and RP105 staining on VSMC (E). RP105 staining in media area of a murine femoral artery (F). Proliferation of VSMC of WT and RP105^−/−^ (G) Liquid-scintillation counting after 16 hours of WT and RP105^−/−^ VSMC. VSMC of show a RP105^−/−^an increased proliferative response to LPS. VSMC were cultured in medium containing 10%FCS or 10%FCS with1 ng/ml LPS. Starved WT and RP105^−/−^ VSMC were used as controls. White bars represent WT VSMC, black bars represent RP105^−/−^ VSMC.

Both TLR4 and RP105 mRNA are expressed in VSMC and as expected overnight incubation with LPS decreased mRNA expression for both TLR4 as described previously on leukocytes [27], [28] as well as RP105 (Figure 1A–B). TLR4 and RP105 protein was detected by immunohistochemistry (IHC) and was also decreased after overnight LPS incubation (Figure1C–D). In non-stimulated cells co-localization between TLR4 and RP105 was sporadically detected (Figure 1E and Figure S1). To detect RP105 expression in the vessel wall IHC was performed on cross sections of murine femoral arteries. RP105 was expressed particularly in the media that consists of VSMC (Figure 1F). Furthermore, RP105^−/−^ VSMC showed an increase in proliferation-index (fold of proliferation increase compared to unstimulated controls) compared to wild type controls upon LPS stimulation (Figure 1G).

### RP105 is Present in the Arterial Wall and is Protective in Neointima Formation

**Figure 2:**
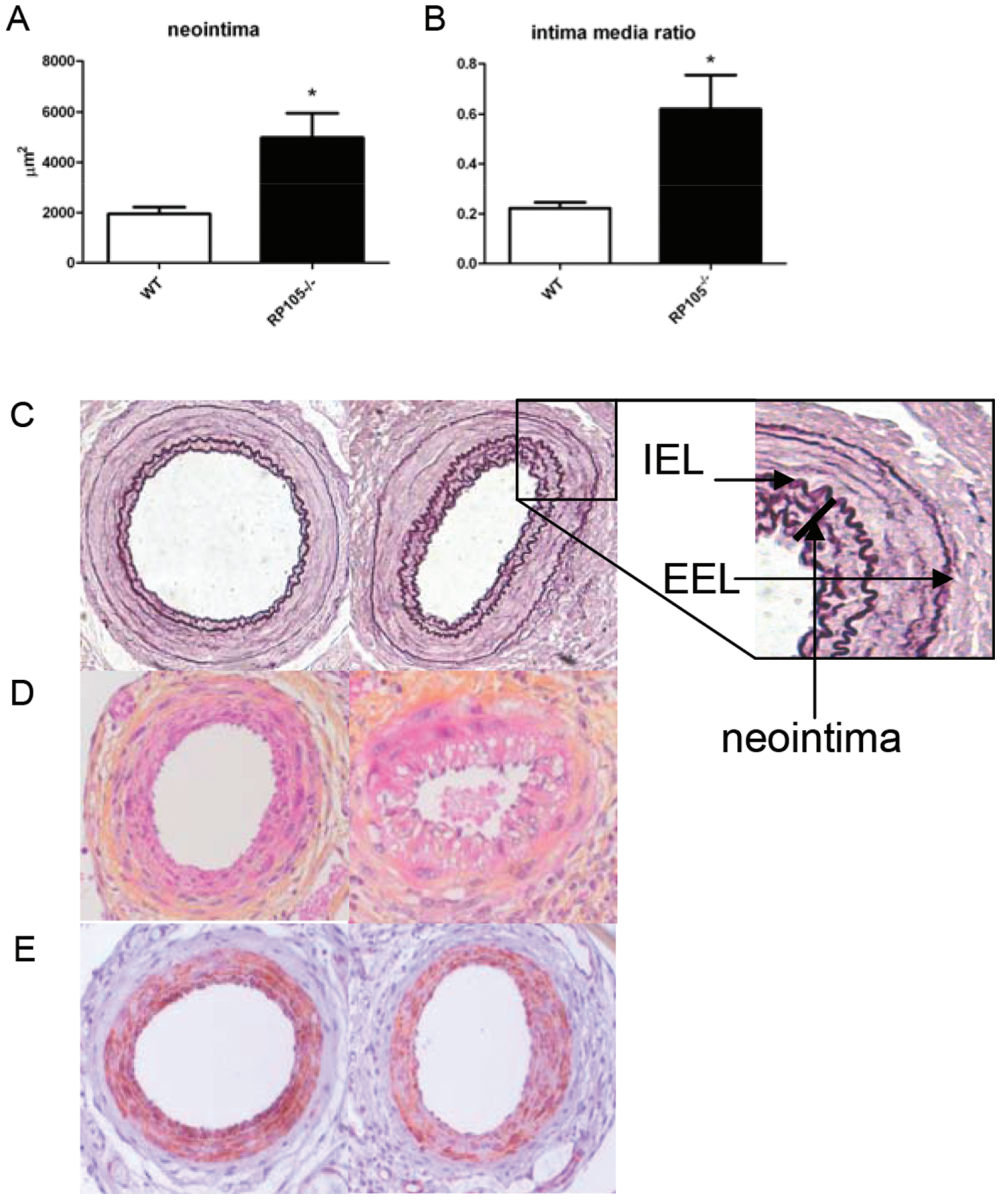
Neointima formation in WT and RP105^−/−^ mice. Neointima formation after femoral artery cuff placement in RP105^−/−^ and wild type mice. Areas of femoral arterial sections were quantified by using 6 sequential sections per segment and are expressed in micrometers squared (mean±SEM). Increased neointima formation in RP105^−/−^ mice compared to WT (wild type) controls (A). Increased intima/media ratio in RP105^−/−^ mice compared to WT controls (B). Representative pictures of Elastin von Giesson (C) HPS (D) and α-smooth muscle cell actin (F) of RP105^−/−^ and WT controls. * = P<0.05 Arrows indicate the Internal Elastic Lamina (IEL) and the External Elastic Lamina (EEL).

Cuff placement induces a mild vascular injury that is driven by a damage induced inflammatory response and can be detected as intimal thickening. Studies to determine the functional role of RP105 in restenosis were initiated. Femoral cuff placement to induce neointima formation was performed in RP105^−/−^ and control wild type (WT) mice. The neointima consists dominantly of VSMC as can be appreciated in (Figure 2AB). Quantification of the neointima revealed that RP105 deficiency resulted in enhanced neointima formation compared to WT mice (4982±974 µm^2^ (n = 8) vs 1947±278 µm^2^ (n = 10), p = 0.0014) (figure 2B). The stronger neointima formation in the RP105^−/−^ caused a difference in intima/media ratio (0.62±0.13 vs. 0.22±0.02, P = 0.0009) (figure 2C). No significant difference was seen in media area size (not shown).

### LPS Induced Increased Neoinitma Formation and Caused Outward Remodeling in RP105 Deficient Mice

**Figure 3:**
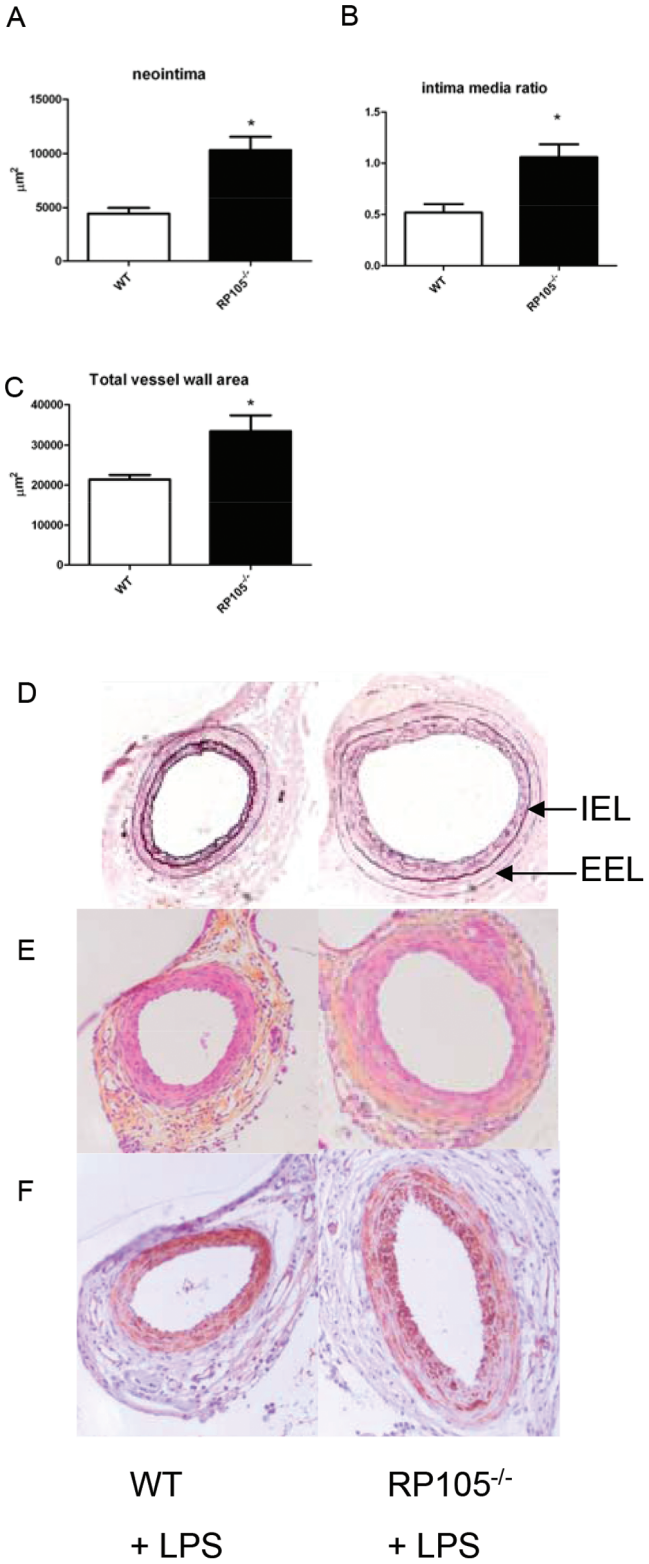
LPS induced neointima formation. Neointima formation after femoral artery cuff placement with LPS in RP105^−/−^ and wild type mice. Areas of femoral arterial sections were quantified by using 6 sequential sections per segment and are expressed in micrometers squared (mean±SEM). Increased neointima formation in RP105^−/−^ mice compared to WT controls after local LPS application (A). Increased intima/media ratio in RP105^−/−^ mice compared to WT controls after local LPS application (B). Increased outward remodeling in RP105^−/−^ mice compared to WT controls after local LPS application (C) Representative pictures of Elastin von Giesson (D), HPS (E) and α-smooth muscle cell actin (F) of RP105^−/−^ and WT controls. * = P<0.05 Arrows indicate the Internal Elastic Lamina (IEL) and the External Elastic Lamina (EEL).

To initiate a stronger TLR4-driven vascular remodeling, the experiment presented above was repeated with local application of the TLR4 ligand LPS (1 µg/µL in pluronic gel) around the cuffed artery. Local LPS application caused augmented neointima formation in both groups (RP105^−/−^ (n = 9) vs WT (n = 8)) and again significantly stronger neointima formation in RP105^−/−^ mice (10316±1243 µm^2^ vs. 4208±555µm^2^, p = 0.0002) (figure 3A). Intima/media ratio after LPS application increased in both groups (1.05±0.12 vs. 0.52±0.07 p = 0.0037) (figure3B). Previously, we showed that TLR4 induced neointima formation via local LPS application caused outward remodeling [4], [18]. Therefore, we compared the total vessel wall areas of both groups to study the outward remodeling response. Outward remodeling in the RP105^−/−^ mice (33315±3945µm^2^ vs. 21401±1140 µm^2^, p = 0.027) (figure 3C) was significantly stronger compared to the controls.

Previously, RP105-mediated suppression of TLR4 signaling by plasmid overexpression in HEK-293 cells was shown *in vitro* [15]. So far we found negative effects of RP105 deficiency on vascular remodeling and therefore we hypothesized that RP105, by regulating TLR signaling, might have therapeutic potential in post-interventional vascular remodeling. Therefore we produced soluble RP105 protein, characterized it and tested its therapeutic potential.

### Soluble RP105 Protein – Characterization

**Figure 4:**
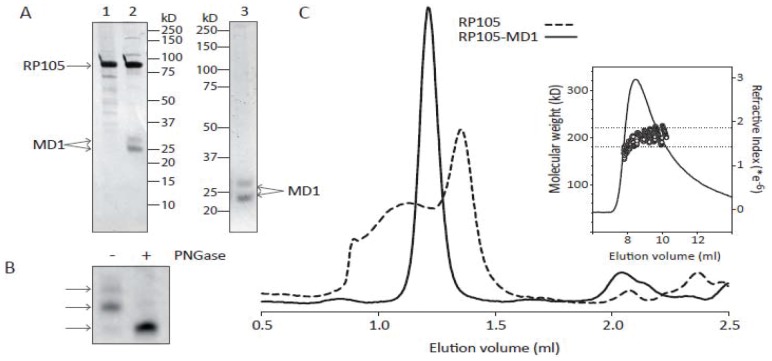
Soluble RP105 protein – characterization. Purification and analysis of RP105, MD1 and RP105-MD1 complex. RP105 (lane 1) and RP105-MD1 complex (lane 2) purified by nickel- and streptactin- tandem affinity purification and analysed by SDS-PAGE (10%) and subsequent silverstaining. Lane 3 shows MD1 purified by nickel-affinity purification and analysed by SDS-PAGE (12%) and subsequent Coomassie staining (A). SDS-PAGE analysis of PNGase deglycosylation of SDS denatured MD1 (B). Size exclusion chromatography (Superdex200 PC3.2/30) of the purified RP105 (dashed line) and RP105-MD1 complex (solid line). Molecular weight determination of the RP105-MD1 complex using SEC-MALS (inset). The dashed horizontal lines indicate a molecular weight of 180 kD and 220 kD, respectively (C).

SDS-PAGE analysis of the purified proteins, shows that the solRP105-MD1 complex and MD1 preparations are >95% pure, whereas the solRP105 preparation shows some impurities (Figure 4A). solRP105 has the expected molecular weight of ∼90 kD whereas MD1 shows as a double band at approximately 23 and 26 kD (Figure 4A). Deglycosylation of denatured MD1 shows that the two bands in the MD1 preparation are due to differential glycosylation of the two N-glycosylation sites at positions Asn96 and Asn156 (Figure 4B). During size-exclusion chromatography, the solRP105-MD1 complex shows a single peak with an elution volume that is consistent with a hetero-tetrameric RP105_2_MD1_2_ complex, as was observed in the recently published crystal structures of the RP105-MD1 complex (16, 18). solRP105 shows a broad peak that is indicative of aggregation with a peak maximum at an elution volume that is consistent with monomeric RP105 (Figure 4C). The molecular weight of the complex was determined more accurately using SEC-MALS and yielding a value of 208±11 kD, which is in good agreement with the expected molecular weight of 214 kD for fully glycosylated RP105_2_MD1_2_ complex.

### Soluble RP105 Protein – Functionality

**Figure 5:**
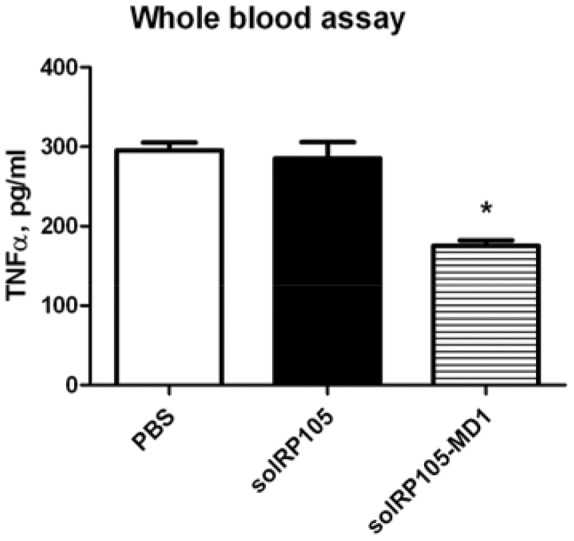
Soluble RP105 protein – functionality. Whole blood stimulation. Blood stimulated with LPS and PBS or combined with purified solRP105 protein or purified solRP105-MD1 protein. Supernatant from triplicates was used for TNFα measurement. * = P<0.05.

We were able to show the increased TLR4 mediated inflammatory response in RP105 deficient myeloid cells (Figure S2). To see whether solRP105 or solRP105-MD1 could have effects on cytokine production by these circulating cells we performed an *ex vivo* whole blood stimulation assay in the presence of these proteins. Blood was incubated with LPS in the presence of solRP105, solRP105-MD1 complex or PBS and TNFα was after measured after overnight LPS stimulation. solRP105-MD1 showed a marked decrease in TNFalpha production compared to controls (Figure 5).

### solRP105-MD1 Reduced Neointima Formation

**Figure 6:**
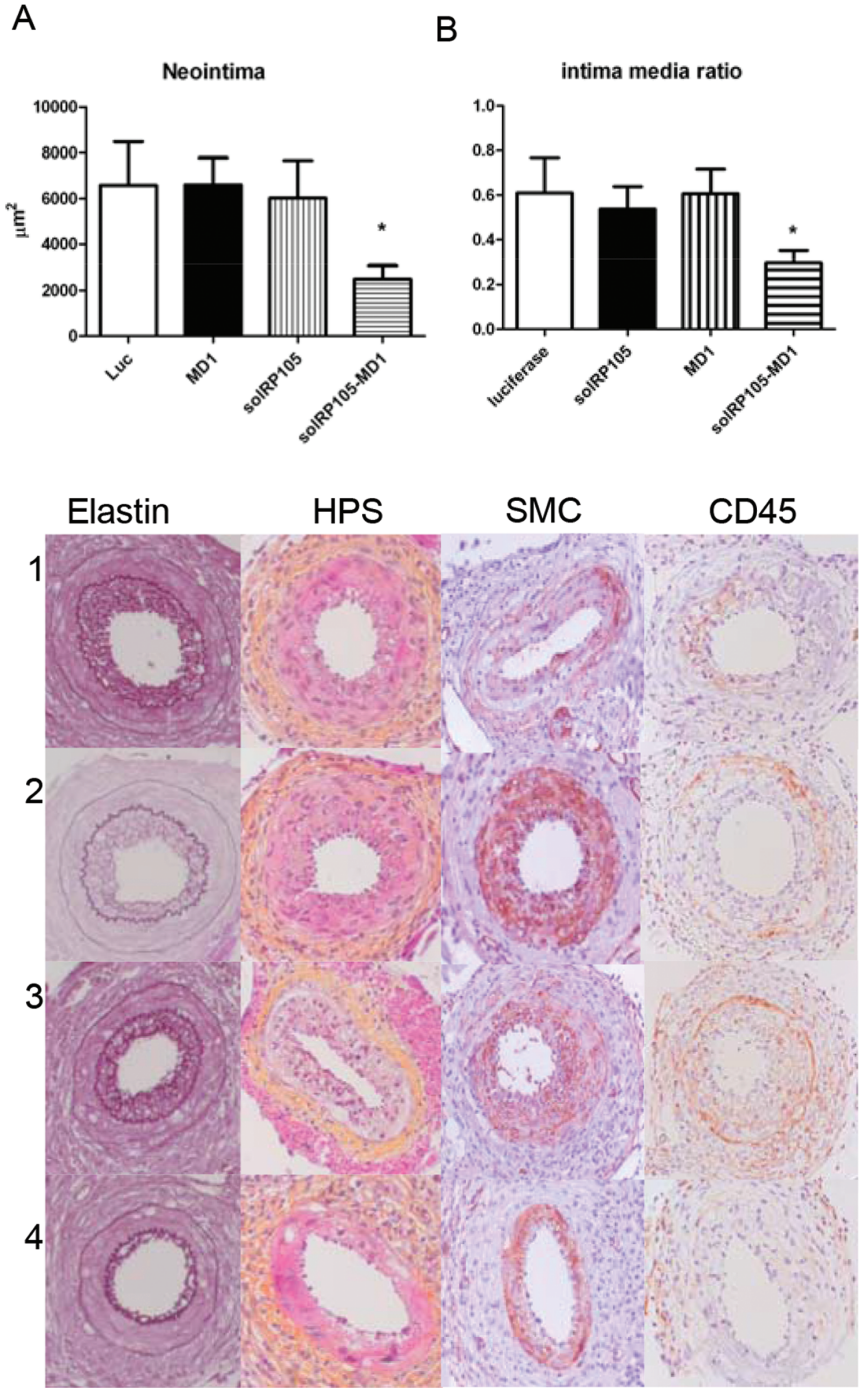
SolRP105-MD1 reduced neointima formation. Neointima formation after femoral artery cuff placement with LPS in hypercholesterolemic APOE3Leiden mice. Areas of femoral arterial sections were quantified by using 6 sequential sections per segment and are expressed in micrometers squared (mean±SEM). Decreased neointima formation in mice that had overexpression of solRP105-MD1 (A). Intima Media ratio (B) Pictures of Elastin von Giesson (C) HPS (D) α-smooth muscle cell actin (E) and CD45 (F) of Luc(1) solRP105 (2) MD1 (3) solRP105-MD1 (4) * = P<0.05.

To see whether solRP105 could be a functional inhibitor of neointima formation in a more humanized model with mild foam cell formation in the neointima we applied our cuff model in ApoE3Leiden mice. Since RP105 also had strong effects on macrophages [15] important in foam cell formation [25] and the above described effects on VSMC proliferation we hypothesized that overexpression of the soluble form of the RP105 receptor, either alone or in complex with MD1, could have potential therapeutic benefits to reduce restenosis. Plasmids encoding solRP105, MD1 or luciferase (control) were injected in calf muscles followed by electroporation into the muscle cells 3 days before cuff placement in transgenic ApoE3Leiden mice that develop a diet induced hypercholesterolemia. This resulted in systemic overexpression of the recombinant proteins, as we have demonstrated previously [8], [29], [30]. Overexpression of both solRP105 and MD1 proteins combined caused a significant reduction in neointima formation compared to luciferase control (2500±573 vs. 6581±1894 µm^2^, P<0.05) and a more beneficial intima media ratio (Figure 6A–D), whereas overexpression of either solRP105 or MD1 did not affect neointima formation.

## Discussion

To our knowledge, the present study is the first to describe a role of RP105 in vascular disease. RP105 is expressed in cultured VSMC, as well as in the media. Furthermore, RP105 deficient mice develop less neointima after vascular injury and in these mice lacking the inhibitor TLR4 inhibitor R105, TLR4 activation via LPS clearly enhanced vascular remodeling more profoundly. Finally we demonstrated that overexpression of a soluble RP105/MD1 protein complex strongly reduced neointima formation.RP105 can be distinguished from other endogenous TLR regulators by being an extracellular TLR4 structural homolog with contrasting capabilities like the inhibitory effects on myeloid cells and activation of B cells [13]. Our study shows that RP105 protein is also expressed by vascular smooth muscle cells (VSMC). Others have shown RP105 presence on smooth muscle cells of the airway [31]. Additionally, we found via immunostaining as well as via RT-PCR that the expression of both receptors is down regulated upon overnight incubation with LPS as can be appreciated in figure 1. This is in line with previous observations in monocytes/macrophages exposed to LPS, which show reduced responses to second stimulation with LPS. Furthermore, after an initial LPS stimulation, TLR4 mRNA expression was significantly decreased within a few hours and remained suppressed over 24 h. TLR4 presence at the cells surface rapidly decreased within 1 hour, with a gradual decrease after that [28]. In patients a similar situation can be observed where a reduction in TLR4 expression can be appreciated on monocytes at the end of cardiopulmonary bypass procedures [27]. While TLR4 might be down-regulated as a protective response, RP105 may just follow TLR4 expression as RP105 expression mimics that of TLR4 in human and mouse myeloid cells [15].

RP105 deficiency on VSMC caused an increase in proliferation upon incubation with TLR4 ligand LPS. A stimulating effect of TLR4 on VSMC proliferation was published previously [9]. This points towards a functional role of RP105 in vascular remodeling via its TLR4 inhibitory function, via its effects on VSMC in the pathophysiological process of neointima formation, as a common feature of restenosis. Femoral cuff placement in mice allows employing of a mild mechanically-induced inflammatory-based neointima formation. Lesions in mice consist primarily of VSMC and application of this model to RP105^−/−^ and WT control mice leads to an increase in neointima formation in the RP105^−/−^ mice. This underscores the suggestion that RP105 is also functionally involved in the inhibition of post-interventional neointima formation, a process known to be regulated by TLR4 activation [4], [5] and may play a role in the pathophysiological process of restenosis.

The influence of inflammation on restenosis is most prominent in the early phase after intervention. Interestingly, TLR4 was previously described to be involved in cardiovascular remodeling [5], [6], [32] and local application of LPS causes a strong increase in neointima formation after cuff placement [4]. RP105 deficient mice have a stronger pro-inflammatory response upon systemically delivered TLR4 ligand LPS [14]. In figure 3 we show that short term activation of TLR4 directly after cuff placement via local application of LPS causes a strong increase in neointima formation. As a result, the vessel seems to respond by compensatory outward remodeling to secure the vessel patency [33]. In TLR4-deficient mice this response is inhibited upon local LPS application [4]. Due to a more prominent TLR4 activation in RP105^−/−^ mice we see that these mice have an increase in outward remodeling.

So far a protective role for RP105 on post-interventional remodeling is appreciated. This indicates potential for new strategies to use RP105 in a therapeutic approach. Therefore we focused on the development of recombinant protein expression. Divanovic et al showed that the extracellular region of RP105 is functional only when co-expressed with MD1 [15]. Similar to TLR4 surface expression that is dependent on MD2, RP105 surface expression was shown to be dependent on MD1 [34]. While we could express the soluble ectodomain of RP105 in the absence of MD1, we find that MD1 is necessary to prevent its aggregation. Monomeric RP105 protein is not capable of inhibiting the LPS response on whole blood while the RP105-MD1 complex did inhibit LPS induced TNFα production, a factor known to be important in neointima formation [7]. The exact mechanism of how RP105-MD1 regulates TLR4 signaling is still under discussion. However, it is suggested that the complex formation is crucial, since monomeric RP105 is highly unstable. It has been shown that RP105-MD1 interacts directly with TLR4-MD2 [15]. The unusual 2∶2 homodimer of the RP105-MD1 complex suggests two possible mechanisms for inhibition of TLR4-MD2 [18]; either (i) a lateral binding of TLR4-MD2 to the RP105-MD1 complex which would involve direct MD1-MD2 interactions or, (ii) the formation of quasi-symmetrical TLR4-MD2/RP105-MD1 complexes reminiscent of the usual ligand-induced TLR homodimers. In both scenarios RP105-MD1 could block TLR4 homodimer formation and possibly prevent/destabilize LPS binding to MD2 [18]. However, the second mechanism was considered to be more plausible since it explains the regulation of the LPS response by RP105–MD1 regardless of LPS binding [18].

To set more focus on the therapeutic potential we initiated experiments to study the therapeutic potential of RP105 in a more humanized model having vessel damage combined with a diet induced hypercholesterolemia, i.e. cuff induced neointimal formation in hypercholesterolemic ApoE3*Leiden mice. No effect was seen of gene transfer of vectors expressing MD1 as a single factor. The absence of an effect of MD1 expression, as was also previously shown in vitro [15], demonstrates that the mechanism that leads to reduction in TLR4 signaling does not rely on MD1-MD2 interactions, thus the above-discussed scenario 1 is unlikely.

Overexpression of solRP105 alone as a single factor did not alter neointima formation suggesting that the endogenously produced MD1 is not sufficient to stabilize RP105 by formation of complexes as is required in vitro (Figure 4). Combined overexpression of both proteins, however, resulted in the formation of a functional complex and led to a strong decrease in neointima formation. The soluble RP105/MD1 complex may act as a decoy receptor and as such have a therapeutic potential.

Apparently, overexpression in vivo of solRP105 with MD1 at the site of production is essential to form a stable and effective RP105-MD1 complex for functional inhibition of neointimal formation. These results are also supportive for the quasi-symmetrical working mechanism of RP105-MD1, the above-discussed second scenario of RP105 mediated TLR4 inhibition.

In summary, we demonstrate the presence of RP105 on cultured VSMC and in VSMC in vivo in media and neointima and the increased restenosis in RP105^−/−^ mice indicate RP105 as a mediator of restenosis, probably as an inhibitor of TLR4. Furthermore, solRP105 significantly modulates the strong TLR4 driven vascular remodeling. Thus RP105 is an interesting new factor involved in the complex regulation of vascular remodeling and can be used to develop novel therapeutic strategies to inhibit neointima formation.

## Supporting Information

Figure S1 **Colocalized TLR4 and RP105 staining on VSMC shown by confocal microscopy.**
(PDF)

Figure S2 **TNFα levels produced upon LPS stimulation by dendritic cells (A) and macrophages (B) from WT and RP105^−/−^ mice.** * = P<0.05.
(PDF)
