# Supplementary figures and images for: TLR Accessory Molecule RP105 (CD180) Is Involved in Post-Interventional Vascular Remodeling and Soluble RP105 Modulates Neointima Formation
Source: PLoS One. 2013 Jul 2;8(7):e67923. doi: 10.1371/journal.pone.0067923 (PMC3699456; doi:10.1371/journal.pone.0067923)

Figure S1

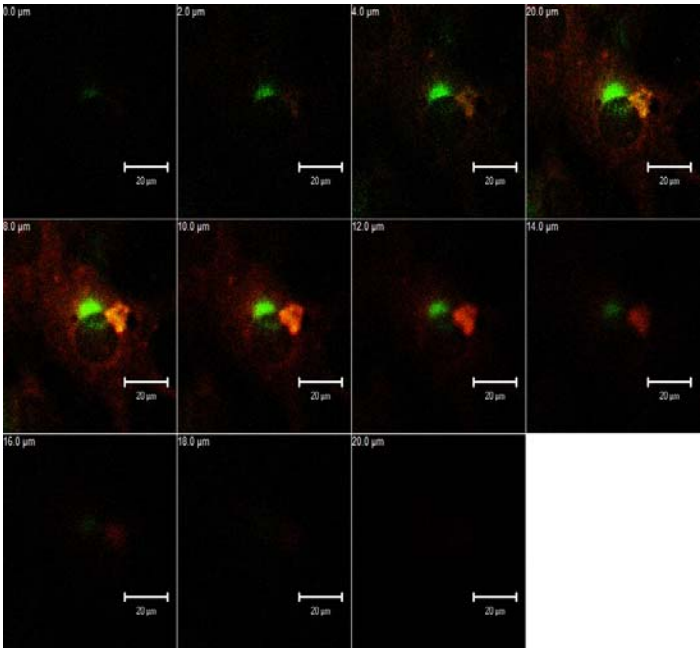

Supplement: Figure S1 — Colocalized TLR4 and RP105 staining on VSMC shown by confocal microscopy. (PDF) [file pone.0067923.s001.pdf]

Figure S2

A

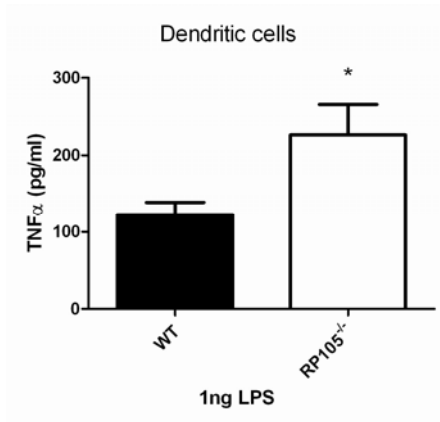

B

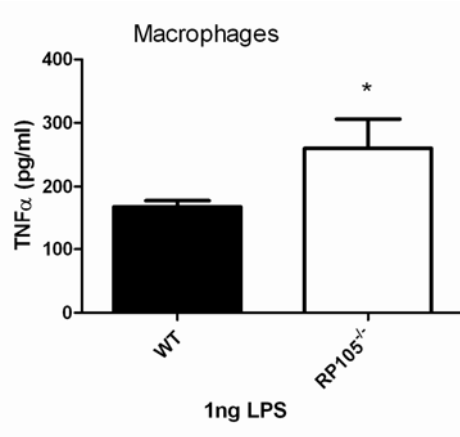

Supplement: Figure S2 — TNFα levels produced upon LPS stimulation by dendritic cells (A) and macrophages (B) from WT and RP105−/− mice. * = P<0.05. (PDF) [file pone.0067923.s002.pdf]
